# Breast Cancer messaging in Vietnam: an online media content analysis

**DOI:** 10.1186/s12889-020-09092-8

**Published:** 2020-06-19

**Authors:** Chris Jenkins, Dinh Thu Ha, Vu Tuyet Lan, Hoang Van Minh, Lynne Lohfeld, Paul Murphy, Le Thi Hai Ha

**Affiliations:** 1 Centre for Public Health, Queen’s University Belfast, Belfast, UK; 2 Hanoi University of Public Health, Hanoi, Vietnam; 3 Institute of Anthropology, Hanoi, Vietnam; 4 School of Arts, English and Languages, Queen’s University Belfast, Belfast, UK

**Keywords:** Breast Cancer, Vietnam, Media content analysis, Health communication, Global Health, Noncommunicable diseases (NCDs)

## Abstract

**Background:**

Breast cancer incidence is increasing in Vietnam with studies indicating low levels of knowledge and awareness and late presentation. While there is a growing body of literature on challenges faced by women in accessing breast cancer services, and for delivering care, no studies have sought to analyse breast cancer messaging in the Vietnamese popular media. The aim of this study was to investigate and understand the content of messages concerning breast cancer in online Vietnamese newspapers in order to inform future health promotional content.

**Methods:**

This study describes a mixed-methods media content analysis that counted and ranked frequencies for media content (article text, themes and images) related to breast cancer in six Vietnamese online news publications over a twelve month period.

**Results:**

Media content (*n* = 129 articles & *n* = 237 images) sampled showed that although information is largely accurate, there is a marked lack of stories about Vietnamese women’s personal experiences. Such stories could help bridge the gap between what information about breast cancer is presented in the Vietnamese media, and what women in Vietnam understand about breast cancer risk factors, symptoms, screening and treatment.

**Conclusions:**

Given findings from other studies indicating low levels of knowledge and women with breast cancer experiencing stigma and prejudice, more nuanced and in-depth narrative-focused messaging may be required.

## Background

Given the increasing incidence and growing burden of breast cancer in Vietnam, and low levels of knowledge and awareness concerning breast cancer in the general population [1–7], we sought to: map media content in Vietnam; present findings from a content analysis (rank-ordered frequency counts) of common themes; and conduct an inductive qualitative analysis on media content and images. Low levels of knowledge and awareness of breast cancer symptoms and treatment options continues contribute to late diagnosis of breast cancer across low and middle income countries (LMICs [8]) [9–11].

The media is crucial within health communication. It is a diverse and constantly evolving industry with multiple stakeholder groups and interests, and can be information-based, entertainment-orientated, or a combination of the two. It can be for-profit, or publically owned, and is utilised by governments, commercial entities, charitable organisations, and individual stakeholders. Media often has a reach that greatly exceeds that of academic publications [12]. It may be used to promote health messages, while equally it may be used as a vehicle for the proliferation of misinformation about health.

In her analysis of 10 years of health content in the media (via international communications journals published in English), Kimberly N. Kline writes, “research consistently has concluded that health-related content in popular media is problematic from a health promotional standpoint.. (and) that media representations are still suspect – fraught with inaccuracies, misleading and problematic themes, and images that stereotype and stigmatise” [13]. This nexus between the potential benefits that may ensue from well-targeted and delivered health messaging through the media, and the contrasting potential for the proliferation of misinformation on health, makes media an important area of study in global and public health.

Detailed information on readership and audience of different Vietnamese news outlets, including our chosen sources, is limited. Media engagement is, however, estimated to be high. An estimated 84% of young people aged 18–29 read the news online at least once per day, compared to 51% of 30–49 year olds and 10% of those aged 50 and above [14]. The International Telecommunications Union estimates 70.4% of the Vietnamese population uses the internet, with a high number of people having mobile phone subscriptions (147 subscriptions per 100 of the population) [15]. Adult literacy is high in Vietnam, reported as 94% in 2009 [16]. Social media use in Vietnam is high, particularly among young and higher income groups, and many people use social media as an access point for more traditional news sources. This study focused predominately on traditional media (newspapers), but sampled from their online platforms. Social media often links directly to these platforms, and as such a binary distinction between ‘old’ and ‘new’ media may over simplify how users move between different media platforms.

The aim of this study was to investigate and understand the content of messages concerning breast cancer in online Vietnamese newspapers. The study investigated what was being shared (for example, textual content and images); how it was shared (e.g. testimonies, statistics etc.); the level of accuracy; and what could be learned about how breast cancer is represented within Vietnam. The study aims to contribute knowledge that will be of use in the design of health promotional content, specifically in relation to breast cancer. Few media content analyses have been conducted in relation to breast cancer in LMICs. Studies that have engaged with this topic tend to be focused on high-income contexts [17–19]. No media content analysis on breast cancer in Vietnam has been previously published.

## Methods

Media content analysis is a broad umbrella term for the analysis of different elements of the media in relation to a particular subject. A significant amount of work has been published on its application within health [13, 20–23]. This work focuses on systematic thematic coding of the textual content of articles based on quantitative measures of the presence or absence of a theme.

In this study, data was sourced over a 12 month period between 1 August 2017 and 31 July 2018. Online platforms of each news source were searched using the terms ‘breast cancer’ and ‘cancer’ (Vietnamese search terms of ‘ung thư vú’ and ‘ung thư’). This returned 225 articles. We then added the criteria that the term “breast cancer” had to appear at least 2 times in the content. This returned 172 articles for full text screening. Forty-three articles were rejected because they were not relevant to breast cancer and our study objectives. One hundred twenty nine articles were included in this analysis. Online news sources were chosen because they provide easily accessible and archived news content that appears both online and in print. Inclusion criteria was any media content including articles, opinion pieces, and images that focused upon or included specific information on breast cancer.

Two Vietnamese researchers on the study team scored the selected articles using a data collection matrix developed collaboratively by the team. The matrix is based on previous work developed by Hilton and colleagues [20] to measure media content on obesity in UK newspapers and was adapted to provide a quantitative overview of content concerning breast cancer in Vietnam (see supplementary information). This matrix allowed standardisation of the dataset in terms of the type of content in each article, headline, word count, and image analysis. Data were given a sore of 0 (No) or 1 (Yes) on whether an article included specific thematic content (e.g. information on symptoms of breast cancer).

The research team further modified this tool to include data collection boxes for key quotations, information from the perspective of individuals cited or referenced in each article, and to code images used in the articles. This tool enabled a comprehensive mixed-methods and hybrid approach to analysis. This approach was deemed to be the most appropriate way of ensuring rigour and deepening our analysis, given the importance of understanding nuances in language, culture and context.

### Sample

Six different news sources were selected to achieve a comprehensive overview of stories and content on breast cancer in Vietnam. These sources were chosen as they consisted of both mainstream and popular general news, and targeted outlets for health and women’s interest and health. The sources were selected collaboratively by the research team and discussed until consensus was reached. Criteria for selection included estimated reach, being a leading publication within a topic area (e.g. health), and diversity of overall sample (in terms of thematic focus and connection to different Governmental departments). The six sources included within our study were: *Dan Tri* (coded in the results section as ‘A’)*, VN Express* (B)*, Vietnam Net* (C)*, The People* (D)*, Health and Life* (E), and *The Women* (F). Under Vietnam’s Press Law (2016) each publication is supported and managed by different ministries within government, as indicated in the Table 1. Publications are regulated under the Press Law (2016), which states that each press-managerial agency has the power to “determine the type of press, guidelines, objectives, target audience, the language of each publication/ press product; determine tasks, operational directions of the press agencies”.

**Table 1:** Sources, Focus and Press-managerial agencies

Source	Link	Press-managerial agency	Focus
VN Express	https://vnexpress.net	Vietnam Ministry of Science and Technology	Popular online news
Vietnam Net	https://vietnamnet.vn	Vietnam Ministry of Information and Communication	Popular online news
Dân trí (Intellectuals)	https://dantri.com.vn	Vietnam Study Promotion Association	Popular online news
Nhân dân (The people)	https://nhandan.com.vn	Vietnam Communist Party	Official news
Sức khỏe và đời sống (Health and Life)	https://suckhoedoisong.vn	Vietnam Ministry of Health	Health specific issues
Phụ nữ (The Women)	https://www.phunuonline.com.vn	Vietnam Women Union	Women specific issues

### Rigour and reliability of data

#### Quantitative component

A standard operating procedure was created to ensure that data were collected in a rigorous and standardised way. Codes were created to allow frequency counting of the data across each article. Definition sheets were created collaboratively with partners in Vietnam, to improve rigour by ensuring that each content code had an agreed meaning, and that there was consensus within the study team as to what each content code related to. Training workshops with data collectors were conducted in which the team practiced how to input data into the matrix, highlighting any problems and confusion and allowing that to be addressed in an immediate manner.

A sample of the data collected was double coded (32 out of 129 articles, 25%) by the two lead Vietnamese researchers on the study team. A Cohen’s Kappa analysis was then conducted to assess the degree of agreement across the different codes. Where the Kappa statistic indicated low agreement, the research team reanalysed data and discussed and resolved discrepancies.

#### Qualitative and image analysis

Members of the research team translated headlines and first paragraphs of each article from Vietnamese into English. Translations were systematically corroborated between the team (a process replicated throughout all stages of translation [24]). From this list, the research team purposively selected a sample of 32 articles to translate in full to use for qualitative analysis. After 32 articles had been analysed, the research team discussed and agreed that saturation had been met [25, 26]. Each article was categorised in relation to its dominant theme (e.g. prevention, screening, nutrition, exercise etc.). Weighted totals were calculated to generate a representative sample (eg, 22% of articles related to prevention, therefore 22% of the 32 selected articles translated also related to prevention). Each article was selected through reading and scoring headlines and first paragraphs.

To analyse qualitative content of full-text translations, a thematic code book was designed collaboratively by the research team and was independently tested by each member of the team on ten randomly selected articles. Open codes [25] were created after each researcher independently read and re-read the transcripts. This data-driven approach process was further informed by DeCuir-Gunby et.al [27], who describes a five-part process for inductive coding: “ [1] reduce raw information [2]; identify subsample themes [3]; compare themes across subsamples [4]; create codes; and [5] determine reliability of codes.” The team discussed whether the code book needed to be refined before being applied to the whole set of 32 fully translated articles. During these conversations, coding categories were added to provide space for data and comments on content in each article (for example, was content presented in positive or negative terms and/or tone) and any data relating to class, gender, regional or other potential biases (code book available in supplementary information). Tone (alarming/reassuring or neutral) was assessed based on individual reading of headlines and content and discussion and agreement within the research team.

The study team (2 Vietnamese researchers and 2 international researchers) independently coded the 32 translated articles, and then discussed and agreed major themes emerging from the data [13]. The identification of key quotes by each member of the team allowed discussion on which themes were most relevant, and what should be included in the analysis.

After a thorough reading of all articles and images, an image analysis framework was created (see supplementary material). This included information on content of the image (medical procedure, information, diet, exercise), individuals in the image (patient, medical professional, celebrities etc.), and whether the image was a stock image or taken for the specific purposes of the article it was included within. When it was not otherwise clear (e.g. when photos were not referenced to a stock image provider), authors made a judgement on whether images were stock images or original and taken for the purposes of the article. Specific images were selected for further analysis and to be used as examples within this paper based on their salience and relevance to wider themes identified within the analysis.

## Results

A total of 129 articles met our inclusion criteria and were included in the analysis. A list of headlines and first paragraphs are included in Table 3 (supplementary information), while frequency count of pre-specified content in each article is presented in Table 2. The following themes were identified from both the quantitative and qualitative data: (i) prevalence of accurate and factual information; (ii) limited information concerning screening; (iii) positive coverage regarding treatment; (iv) emphasis on diet; and (v) limited personal stories or experiences. The headlines were rated as to the tone of each article’s headline. The most of them (*n* = 59, 45.7%) were rated by the team as ‘neutral’; another 42 (32.6%) were rated as ‘reassuring’; and 28 (21.7%) were rated as ‘alarming’. The mean length of articles was 625 words, with a range of 32 to 2857 words.

**Table 2:** Frequency counts of pre-specified content in media article

Content Code	Alarming	Reassuring	Neutral
Rate Headline	28 (21.7%)	42 (32.6%)	59 (45.7%)
**Content Code**			**Coded ‘Yes’**
Mentions treatment options for women with breast cancer in a reassuring tone (ie treatment can be curative)			60 (46.5%)
States breast cancer rates			57 (44.2%)
Correctly identifies risk factors related to breast cancer			51 (39.5%)
Mentions screening options for breast cancer			49 (38.0%)
Encourages women to speak to their healthcare provider if they experience symptoms			45 (34.9%)
Correctly identifies symptoms of breast cancer			43 (33.3%)
Advertises pharmaceutical interventions or products for breast cancer			42 (32.6%)
Causal Factors linked to Westernisation (eg. diet)			35 (27.1%)
Mentions treatment options for women with breast cancer in an alarmist tone (‘deforming’, hair loss)			29 (22.5%)
Provides information on where and how to access screening services			27 (20.9%)
Describes what is involved in a screening procedure			23 (17.8%)
Mentions social support networks for women with breast cancer			23 (17.8%)
Mentions breast cancer incidence as increasing			21 (16.3%)
Blames women for their diagnosis (for delaying speaking to a healthcare provider)			13 (10.1%)
Mentions Government interventions to support women with breast cancer			12 (9.3%)
Mentions social challenges related to breast cancer, such as stigmatisation or experiencing discrimination			11 (8.5%)
Mentions traditional medicine as viable alternative to biomedical interventions			10 (7.8%)
Incorrectly identifies risk factors (eg. karmic beliefs/other)			9 (7.0%)
Mentions women with breast cancer experiencing relationship breakdown with intimate partners			9 (7.0%)
Mentions breast cancer as a cosmetic / beauty problem			8 (6.2%)
Mentions arts-interventions to raise awareness about symptoms of breast cancer			8 (6.2%)
Mentions economic challenges for women with a breast cancer diagnosis			7 (5.4%)
Mentions arts-interventions to reduce stigmatisation of women with breast cancer			7 (5.4%)
Mentions financial catastrophe related to breast cancer treatment			4 (3.1%)
Criticises the health system and healthcare providers for lack of systematic capacity to respond to breast cancer			4 (3.1%)
Incorrectly identifies symptoms of breast cancer			2 (1.6%)
Mentions breast cancer as a burden to the health system			1 (0.8%)
Blames women for their diagnosis (e.g. karmic causes)			0 (0.0%)

### Style and tone

Articles tended be largely medical and neutral in style and tone. Authoritative voices cited in the articles tended to be ‘experts’ (mainly medical professionals). Some of these articles referred to experts generically, for example, “as recommended by doctors, women under 50 years old with thick breast tissue forming a lump should have mammography or ultrasound to screen for cancer” (B049). Other articles referred to information from specific Vietnamese doctors (e.g. E025), whereas a large number of articles referred to statements made by international doctors from the US, Australia, the UK, and Singapore (e.g. C002, B049, A001). Many articles additionally included a comment or advice from the Vietnamese Ministry of Health.

### Prevention, risk factors and symptoms

An analysis of both the quantitative and qualitative data showed a relatively high degree of coverage of factual information on breast cancer incidence rates in Vietnam (44.2% of articles); the correct identification of symptoms (33.3%); and the correct identification of risk factors for breast cancer (39.5%). In contrast, there is very little explicit misinformation circulated within our sample concerning incorrect identification of symptoms (1.6% of articles) and identifying incorrect risk factors (7.0%) (corroborated with information from the Centers for Disease Control and Prevention USA (CDC) and Cancer Research UK websites). Information is largely clear and accurate, as shown in the following excerpts from mass media outlets:

*In order to early detect breast cancer, you must pay attention if there are any lumps on the breast, nipple deformation, fluids coming out of the nipple, redness or abnormal marks on the breast. Consult the doctor if you have any of these symptoms. (B049).*

*Breast cancer doesn’t have any clear symptoms and requires judgement from an Oncology doctor. The symptoms may include: A lump in breast that won’t disappear even after the menstruation is over; a node at the armpit may be a sign of breast cancer metastases to the nearby lymph nodes; changes in size and shape of the breast; change in breast skin and nipple colour as well as nipple excreting strange fluid. (C007).*

Many of the articles used lists as a way of conveying information, for example “8 simple methods to prevent breast cancer” (B048) & “4 common misunderstandings about breast cancer in women” (B049). A large degree of content on prevention focused on the importance of diet, which is discussed in depth later in this paper.

### Media content on screening

More than one third of articles (38.0%) mention screening for breast cancer, however, less than half of these articles (17.8% of the total sample) describe what is involved in a screening procedure. Only 20.9% of articles mention a location where women could access screening services. Many of the articles target their information to both younger and older women, highlighting that women of all ages can be affected by breast cancer.

*In other countries, the average age of being diagnosed with breast cancer is 60–65 years old. However, according to experts, in Vietnam the average age of getting breast cancer is only 40–50 years old. In some cases, the patients are diagnosed when they are still very young. (A010).*

Information on self-examination is a frequently included topic, and many articles provide very detailed instructions on how a breast self-exam should be conducted. For example:

*Breast self-examination at home should be conducted 5–7 days after one’s period is over, as the breast would be very soft, meaning it could be easily and accurately checked. The steps are quite simple, you just need to remove your bra and stand in front of a mirror with decent lighting, and observe the shape of your breasts. Lift your right hand above your head, use your left hand to check your right breast. Press and work around in circle from the nipple to the armpit to see if there’s any abnormalities, then do the same with the other breast. Finally, repeat those steps in the lying down position and see if there’s any fluid coming out of the nipple. (A011).*

*Self-screening is the most important method in breast cancer early detection. Women should make the habit of performing self-screening once every month, best after menstruation as this is the time the breasts are the softest. Continue to perform screening periodically even postmenopausal. (B043).*

Information is additionally given regarding screening procedures such as mammography and ultrasound. Despite a clinical breast examination being more affordable for many women (mammography is expensive, and screening is currently not covered under the health insurance model) very little information is given on scheduling an appointment with a doctor and what is involved in a clinical breast exam:

*As recommended by doctors, women under 50 years old with thick breast tissue forming a lump/tumour should have mammography or ultrasound to screen for cancer. Mammography gives quite accurate results, is simple and cheap, and may identify the tumour before you can feel it by hand. However, in order to determine if it’s breast cancer or not, it may require further tests. (B049).*

### Media content on treatment

Media coverage concerning treatment is largely positive, with more articles discussing treatment in reassuring terms (emphasising curative aspect of treatment) (46.5%) rather than articles discussing treatment in either alarmist terms or focusing on the negative side effects of treatment (focusing on factors such as pain, hair loss, scarring etc.) (22.5%). Equally, the headlines of articles were more likely to be reassuring in their tone (32.6% of articles), rather than alarming and sensationalist (21.7%). Neutral or factual headlines were the most common (45.7%). Examples include the following:

*Many breast cancer patients said they’ve heard scary stories regarding radiotherapy, however, several recent studies mentioned that their experiences were much better. Research on 300 women who had radiotherapy showed that half of them had heard of “scary” stories during the treatment. However, only 2% agreed that the stories were true. In fact, more than 80% of the patients said their experience with radiotherapy was “less fearful” than they expected. (A014).*

*Experts says that an operation to remove the tumour and preserve the breast at an early stage makes the women more confident, results in a faster recovery and fewer complications. It also doesn’t require breast reconstructive surgery and has the same survival rate as removing the whole breast. (A011).*

As with the presentation of information on other stages of the cancer journey, the articles tend to have a factual and detailed approach to describing specific processes. In relation to radiotherapy, one articles describes the process as:

*There is no need for the patient to be anesthetized. A computer-control system will be connected to the tubes which are put on the chest. The system will send a small amount of radiation, or the “radioactive particles” into all tubes at the same time. The patient will be able to feel the particles moving into the tubes, but there wouldn’t be any pains. The doctors will be in the next room and are able to monitor and communicate with the patient through a TV screen. The radiotherapist will remove the tubes after the final radiotherapy session. (C007).*

Coverage about treatment was not universally positive, however, with articles (often focusing on the experiences of non-Vietnamese women) highlighted problems with misdiagnosis, mistreatment, and negative impacts of treatment such as pain and infertility.

*Mixing up test results, the doctors removed both of (name anonymised*^1^) *(USA) breasts, while she didn’t have cancer. (name anonymised) (USA) never thought she would be in such an awkward situation. Having been informed that she has genes causing cancer, the 36-year-old mother excepted to have her uterus and both her breasts removed. Only afterward that she found that she was completely healthy. (B039).*

*Having pregnancy-associated breast cancer, (name anonymised) must have her right breast removed, experience painful chemotherapy sessions as well risk infertility. (B051).*

### Lifestyle, Diet & Exercise

Although diet was not one of the topics screened within our quantitative matrix, following our qualitative analysis we conducted a frequency count on all articles. We found that over one-quarter (27.6%) of the articles explicitly referenced lifestyle factors such as diet in relation to prevention, treatment or recovery:

*In order to reduce the risk of getting breast cancer at a young age, you must recognise the importance of having a healthy diet through including green, fresh vegetables in your daily meal and avoiding substances such liquors or cigarettes. (A010).*

*While the genetic, natural factors could not be changed, women can still prevent the disease through proper diet and doing daily exercises. A diet rich in vegetables, fruits, low fat should be the focus. Green pepper, sweet potatoes, garlic, tea, dark-green vegetables, wild salmon, and nuts are the food women should have daily. They are rich in anti-oxidant, and are able to eliminate abnormal cells, preventing cancer from occur. Soy bean and fermented milk are also among the foods that can prevent breast cancer. (B048).*

*The foods which are the kryptonite of cancer and are good for patients. The 8 meals mentioned in the following article should be remembered if you want to improve your immune system and prevent breast cancer: garlic, orange, mango, salmon, green tea, sweet potatoes, pomegranate, seaweed. (C012).*

While much of the information on a balanced diet, reducing alcohol and maintaining exercise is important, other dietary information is sometimes inflated or exaggerated in terms of benefit/risk or certainty within current research. Example include articles with the following headlines: “High-risk of breast cancer for women lacking Vitamin D” (F005) and “Eating cruciferous vegetables helps decreasing the risk of breast cancer” (E009). Other articles present information in which there is a largely undeveloped evidence-base, for example, “Stay away from bread if you have breast cancer” (C004). Toxicity of food is a common concern in Vietnam and was reflected in the content of some of the articles:

*Reduce contact with toxic environments: toxins exists all around us, even when we can’t see them. Reduce toxin in your body by using clean, organic and natural food. Periodically detox yourselves by: eating vegan food, or drinking juice to detox your liver, kidney, and improve your digestive system (A013).*

### Personal stories and experiences

While there is relatively high coverage of factual information about breast cancer symptoms, screening, diagnosis and treatment options in Vietnam, there is a noticeable lack of personal stories and experiences of women with breast cancer. A minority (*n* = 33, 25%) of the articles we examined mention specific challenges faced by women undergoing breast cancer treatment in Vietnam such as economic challenges (5.4% of articles), experience of financial catastrophe from paying for treatment (3.1%), facing social stigmatisation or discrimination (8.5%). Only 7.0% of articles mention intimate relationship breakdown, and only 6.2% mention the aesthetic impact of treatment and challenges it creates for women. Only a small portion of personal stories captured in our media content search show the importance of these themes. While these stories were fewer in frequency, they were longer and more in-depth than many of the other articles. They also tended to focus on the positives of a woman’s experience and response to breast cancer. One article presented excerpts from an interview with a woman who gave four pieces of advice for anyone women undergoing breast cancer through treatment:

*The first word is spirit. The patient must keep a high spirit, stay happy, not breaking down or feel depressed; the second is exercises; the third is medicine, abiding to the treatment the doctor provided; the fourth is diet, diverse yet balanced in order to stay healthy (F009).*

Another, positive campaign highlighted in Dan Tri, focused on a photographic campaign by designer Li Lam, and the launch of her photographs as part of the “Always a woman to me” campaign. The article again focuses on life after breast cancer, and the maintenance of a positive and healthy attitude and self-perception. Li Lam states:

*The production of the album “Always a woman to me” to honour the mothers and the women in general in hope of communicating the message: In any situations, a woman, even without her hair or breasts, is still a mother, a wife. All women should love and be confident with their looks, and shine in their own way. From which loving themselves and lives will come naturally (F006).*

This type of art and arts-based intervention are an additional mechanism through which women’s stories can be shared, but their lack of coverage within the articles we examined shows another potential area for growth. Significantly, many of the articles identified by our study that did highlight personal stories focused on the experiences of Western women and Western celebrities such as Angelina Jolie, Kylie Minogue, and Shannen Doherty, rather than Vietnamese women, as shown in the previous section regarding misdiagnosis and medical errors for women in the USA and the UK.

Not all stories from women were positive. As in the case of one woman who was interviewed about on her experience of breast cancer, her story was presented specifically to try and raise money to support her treatment costs. She is quoted as stating:

*I just worry that should anything happens to me, who would my mother and daughter rely on? Life is harsh, and I just got tangled in it. The disease causes me pain, but thinking about the future of my family is even more painful. I don’t know if I would have enough money for the coming treatment and operation? (A001).*

### Analysis of images

Only four of the 129 articles in the dataset did not include any images. A total of 237 images were analysed (supplementary file of analysed images available on request). Of these articles, the majority 56% (*n* = 133) were coded as being stock images that were not taken for specific use within the article, but most likely sourced off image databases or archives, while 44% (*n* = 104) were coded as original images taken for the specific purposes of illustrating the article.

There was a relatively high concentration of images featuring white women within the articles, with 21% of images featuring at least one white women in the context of either a patient or a women conducting a self-exam. Of these images, 7% (*n* = 17) featured white people in non-medical photos (e.g. eating or exercising), 5% (*n* = 12) featured white medical professionals and 10% (*n* = 23) featured white Western celebrities. However, this number is skewed by one single article having 20 photos of Western celebrities who had a breast cancer diagnosis.

In comparison, 22% (*n* = 51) of the images featured Vietnamese women who had a breast cancer diagnosis. However, 3 articles accounted for 25 of these images, showing the relatively sparsity and lack of visibility of Vietnamese women across our entire sample. Of these images, 5% (*n* = 12) showed Vietnamese people in non-medical photos (e.g. eating or exercising), and 6% (*n* = 15) featured Vietnamese medical professionals. Only 3% (*n* = 6) featured Vietnamese celebrities with breast cancer. Differences were also shown in relation to images of white women conducting self-exams (5% of images, *n* = 12), compared to Vietnamese women conducting self-exams (0.05%, *n* = 1). Other findings include a relatively large number of photos (mainly stock images) showing food (12%, *n* = 28), and images in the form of medical graphics or scans (8%, *n* = 18).

## Discussion

The media may have profound effects not only on how the public conceptualises illness and health, but also on health behaviours [18], thus making it an important area for study. Research conducted on health content in the media has shown that the presentation, accuracy, and communication of health information is often problematic [13].

This paper has sought to investigate some of these themes through gathering and analysing data on newspaper stories and accompanying images that shape popular understandings of breast cancer in Vietnam. Findings show that information about breast cancer in Vietnam tends to be factual. There does not appear to be widespread proliferation of misinformation in the mainstream media. Diet and nutrition are important factors associated with how health is conceptualised in Vietnam, and many articles put a heavy emphasis on the role of diet in both the prevention and treatment of cancer. Additionally, there is a lack of specific information on screening, including how to do a breast self-examination, and a lack of visibility regarding the lived experience of women with breast cancer in Vietnam. Such stories may offer the potential to increase awareness and normalise discussions on breast health.

A question raised by this media content analysis concerns the disconnection between information shared in the media regarding breast cancer risk factors, symptoms and expectations for treatment, and reported knowledge by women in other studies. Despite the largely factual and accurate reporting about breast cancer in the mass media, knowledge and awareness about breast cancer is generally poor [4, 28–30]. This raises questions about the medium, focus, and delivery of content. It also highlights the limitations of mainstream media in communicating health promotional messages to at-risk populations.

A particular gap in the media content is the lack of stories focusing on the nuanced, complex circumstances and stories of Vietnamese women’s experiences of breast cancer. Our analysis of the type and style of media stories on breast cancer in Vietnam, revealed that most articles fall into the ‘Transmission model’ of media communication [31], in that they convey a simple, factual and linear message to communicate information that can improve knowledge. However, increased knowledge in itself is not sufficient to change behaviours and reduce the impact of breast cancer, namely, better detection through self-breast examination, attending screening programmes, and early detection. These findings suggest the value of working with newspaper editors to discuss why publishing more nuanced and personal stories from Vietnamese women may be helpful. The Cultural Model developed by Lewis and Lewis [23] in which meanings are produced, contested and reproduced, and in which a more robust conversation about how breast cancer is experienced and understood, could be integrated into new approaches. Narrative approaches have been shown in some randomised controlled trials (RCTs) to be more effective than informational approaches to changing beliefs and behaviours on breast cancer in other contexts [32].

The simultaneous recognition of the difficult impact of a breast cancer diagnosis on the individual, along with role modelling and the presentation of women as strong, valued, and important in spite of their diagnosis, could be further developed by the media, and may be useful in helping women overcome fears of stigmatisation and the aesthetic impacts of treatment. A relative lack of stories and content featuring Vietnamese women specifically suggests a lack of visibility for individual women and their experiences, and represents an area for potential growth and focus. Sharing more women’s experiences in a positive and supportive way has untold possibilities to build a space in Vietnamese society and families that is fully inclusive of women facing the struggles of a breast cancer diagnosis and its aftermath.

Results of our analysis of images showed a similar relative lack of visibility of Vietnamese women, particularly images of women conducting breast self-exams. The conservative nature of Vietnamese culture may explain the preference of news organisations to use images of white women, particularly if the image reveals intimate areas of the body. As highlighted by Kline and by McGannon, conservative gendered expectations and norms may explain the lack of images of Vietnamese women [13, 18]. ‘Othering’ was also expressed in the images of white women analysed, given their stylized, airbrushed, and sexualised nature. Historically, cancer has been seen as a foreign disease, associated with high-income and Western countries. The extent to which the Vietnamese media perpetuates this idea is possibly revealed in this study. Breast cancer remains something that is largely ‘othered’ through a high concentration of stories on Western women and Western celebrities. Such othering may contribute to Vietnamese women not feeling as if the content presented in the media is directly applicable to them, and thus the behaviours of self-examination and speaking to healthcare care providers should they experience symptoms being not relevant.

Regarding the media content on screening, more articles focused on mammography than clinical breast examinations. Given that clinical breast examination is more accessible and affordable for many women than a mammography, research should be conducted on whether women know what to expect from a clinical breast examination, and what factors may prevent or discourage them from seeking an examination.

This paper contributes important information that may inform the development of future health promotional interventions in Vietnam. It describes what breast cancer content currently exists in the media, identifies gaps in media content, and will help to reduce replication of information in future campaigns. For non-Vietnamese speaking international researchers, it provides an insight into health-related media useful for researchers interested in both breast cancer and a wider range of health concerns. For a wider audience, this paper provides a methodological example and a justification for undertaking media content analysis, particularly for multi-lingual teams interested in co-developing future health promotional activities using mass media. It emphasises the importance of understanding the local media landscape before initiating health promotional interventions in order to improve design of campaigns and avoid replicating existing content.

The descriptive nature of this research may represent a limitation, and future analyses may wish to use other approaches such as framing analysis to better understand representation of breast cancer in the media.

## Conclusion

This study attempts to provide valuable data and insights about the popular context of how breast cancer is presented in the Vietnamese media. Although information is largely accurate, there is a marked lack of stories about Vietnamese women’s personal experiences. Such stories could help bridge the gap between what information about breast cancer is presented in the Vietnamese media, and what women in Vietnam understand about breast cancer risk factors, symptoms, screening and treatment. Particularly for international researchers working in Vietnam, this study describes and analyses current breast cancer media content in Vietnam allowing for well-informed development of future interventions.

## Supplementary information

**Additional file 1: Table 3.** Titles and lead paragraphs of each item included within the media content analysis. A = Dan Tri; B = Vietnam Express; C = Vietnam Net; D = The People; E = Health and Life; F = The Women.

**Additional file 2.** Other supplementary information provided was 1. data collection matrixes and 2. image analysis matrix.

## Data Availability

The datasets used and/or analysed during the current study are available from the corresponding author on reasonable request.

## Abbreviations

CDC: Centers for disease control and prevention, USA
LMICs: Low and middle income countries
NCDs: Noncommunicable diseases
RCT: Randomised controlled trial

## Footnotes

1 Author’s edit. Names not anonymised in original Vietnamese articles

## References

[1] Jenkins C, Minh LN, Anh TT, Ngan TT, Tuan NT, Giang KB (2018). Breast cancer services in Vietnam: a scoping review. Glob Health Action.

[2] MoH, Vietnam (2015). National Strategy for control of NCDs.

[3] Hinh ND, Minh HV (2013). Public health in Vietnam: scientific evidence for policy changes and interventions. Glob Health Action.

[4] Lan NH, Laohasiriwong W, Stewart JF, Tung ND, Coyte PC. Cost of treatment for breast cancer in Central Vietnam. Glob Health Action. 2013;6(1). 10.3402/gha.v6i0.18872 PMC3564971 23394855

[5] Trieu PDY, Mello-Thoms C, Brennan PC (2015). Female breast cancer in Vietnam: a comparison across Asian specific regions. Cancer Biol Med.

[6] Thuan TV, Anh PT, Tu DV, Huong TT. Cancer Control in Vietnam. Where are we? Cancer Control. www.cancercontrolinfo; 2016.

[7] Jenkins C, Ngan TT, Ngoc NB, Phuong TB, Lohfeld L, Donnelly M (2019). Strengthening breast cancer services in Vietnam: a mixed-methods study. Glob Health Res Policy.

[8] World Bank. New country classifications by income level: 2019–2020 [Internet]. 2019 [cited 2020 Mar 20]. Available from: https://blogs.worldbank.org/opendata/new-country-classifications-income-level-2019-2020.

[9] Ginsburg O, Badwe R, Boyle P, Derricks G, Dare A, Evans T (2017). Changing global policy to deliver safe, equitable, and affordable care for women’s cancers. Lancet.

[10] Sullivan T, Sullivan R, Ginsburg O (2015). Screening for Cancer: considerations for low- and middle-income countries. In: Cancer: disease control priorities [internet]. Third edition.

[11] WHO. Breast Cancer Prevention and Control [Internet]. WHO Press. World Health Organisation; 2019 [cited 2019 Sep 20]. Available from: https://www.who.int/cancer/detection/breastcancer/en/index3.html.

[12] Remler D. Are 90% of academic papers really never cited? Reviewing the literature on academic citationst. LSE Impact Blog: London School of Economics and Political Science; 2016. Available from: http://blogs.lse.ac.uk/impactofsocialsciences/2014/04/23/academic-papers-citation-rates-remler/.

[13] Kline KN (2006). A decade of research on health content in the media: the focus on health challenges and sociocultural context and attendant informational and ideological problems. J Health Commun.

[14] Pew Research Centre. Publics Globally Want Unbiased News Coverage, but Are Divided on Whether Their News Media Deliver [Internet]. 2018. Available from: https://www.pewresearch.org/global/wp-content/uploads/sites/2/2018/01/Publics-Globally-Want-Unbiased-News-Coverage-but-Are-Divided-on-Whether-Their-News-Media-Deliver_Full-Report-and-Topline-UPDATED.pdf.

[15] ITU. Viet Nam Profile. 2018.

[16] UNESCO Office for Statistics. Literacy rate, adult total (% of people ages 15 and above) [Internet]. World Bank; 2019 [cited 2019 Sep 10]. Available from: https://data.worldbank.org/indicator/se.adt.litr.zs?end=2018&start=1970.

[17] Andsager JL, Powers A (1999). Social or economic concerns: news and women’s magazines and breast cancer in the 1990s. J, MC Q.

[18] McGannon KR, Berry TR, Rodgers WM, Spence JC (2016). Breast cancer representations in Canadian news media: a critical discourse analysis of meanings and the implictions for identity. Qual Res Psychol.

[19] Post L. Representing disease: an analysis of breast cancer discourse in the south African press. MSc Dissertation at LSE Department of Media and Communications 2014;.

[20] Hilton S, Patterson C, Teyhan A (2012). Escalating coverage of obesity in UK Newspapers: the evolution and framing of the “Obesity Epidemic” from 1996 to 2010. Obesity (Silver Spring).

[21] Hansen A, Cottle S, Negrine R, Newbold C (1998). Mass communication research methods.

[22] Bradley Wright K, Sparks L, O’Hair DH. Health communication in the 21st century. Second ed. New York: Wiley Blackwell; 2013.

[23] Lewis B, Lewis J (2015). Health communication: a Media & Cultural Studies Approach.

[24] Green J, Thorogood N (2009). Qualitative methods for Health Research.

[25] Strauss A, Corbin J (1998). Basics of qualitative research: techniques and procedures for developing grounded theory.

[26] Saunders B, Sim J, Kingstone T, Baker S, Waterfield J, Bartlam B (2018). Saturation in qualitative research: exploring its conceptualization and operationalization. Qual Quant.

[27] DeCuir-Gunby JT, Marshall PL, McCulloch AW (2010). Developing and using a codebook for the analysis of interview data: an example from a professional development research project. Field Methods.

[28] Kim JG, Hong HC, Lee H, Ferrans CE, Kim E-M (2019). Cultural beliefs about breast cancer in Vietnamese women. BMC Womens Health.

[29] Pham T, Bui L, Kim G, Hoang D, Tran T, Hoang M (2019). Cancers in Vietnam—burden and control efforts: a narrative scoping review. Cancer Control.

[30] Jenkins C, Ngan TT, Ngoc NB, Hien HT, Anh NH, Lohfeld L, et al. Experiences of accessing and using breast cancer services in Vietnam: a descriptive qualitative study. BMJ Open. 2020;10(3) Available from: https://bmjopen.bmj.com/content/10/3/e035173. 10.1136/bmjopen-2019-035173 PMC7202702 32209632

[31] McQuail D, Windahl S (2013). Communciation models for the study of mass communications.

[32] Kreuter MW, Holmes K, Alcaraz K, Kalesan B, Rath S, Richert M (2010). Comparing narrative and informational videos to increase mammography in low-income African American women. Patient Educ Couns..

