# Supplementary material for: Breast Cancer messaging in Vietnam: an online media content analysis
Source: BMC Public Health. 2020 Jun 19;20:966. doi: 10.1186/s12889-020-09092-8 (PMC7304170; doi:10.1186/s12889-020-09092-8)
Supplement: Supplementary file 1 — Additional file 1: Table 3. Titles and lead paragraphs of each item included within the media content analysis. A = Dan Tri; B = Vietnam Express; C = Vietnam Net; D = The People; E = Health and Life; F = The Women. [file 12889_2020_9092_MOESM1_ESM.docx]

**Supplementary Information (to be published separately if required)**

**Table 3:** **Titles and lead paragraphs of each item included within the media content analysis.**

A = Dan Tri; B = Vietnam Express; C = Vietnam Net; D = The People; E = Health and Life; F = The Women.

| **Code** | **Title** | **Lead Paragraph** |
| --- | --- | --- |
| A001 | The desperate situation of women with breast cancer | Not only poor, now she also got breast cancer, which makes things even more difficult. Even though suffering from the disease, she still has to take care of her mother whose legs were amputated due to diabetes complications and her daughter who is going to go to university |
| A002 | Risk of breast cancer due to being too thin earlier in life | A recent research found that being underweight when young will increase the risk of breast cancer when premenopausal |
| A003 | Is it possible to “seal” the protein in order to prevent breast cancer? | Scientists have found that by preventing some proteins from reacting with h progesterone (sexual hormone to maintain pregnancy), it is possible to prevent breast cancer among people carrying the high-risk genes |
| A004 | Breakthrough: An injection may “disintegrate” the tumor of terminal cancer | A woman who was predicted to has only few months left to live by doctors, after all breast cancer treatments had failed, was cured completely after a breakthrough injection |
| A005 | Almost 95 million goes to Ms. Hue who has breast cancer | With her husband passed away long time ago, Ms. Hue has to struggle on her own to raise two children. Unfortunately, recently she was also diagnosed with metastasis breast cancer, hence was hospitalized for treatments with many difficulties still lie ahead |
| A006 | Is it necessary for female breast cancer patients at early stage to receive chemotherapy | Most women with breast cancer at earlier stage don’t need chemotherapy post-surgery. This result was published on 3/6/2018 by the breast cancer treatment piloting program funded by the US Government in USA and 5 different countries |
| A007 | Is the increase of CA 15-3 the sign of breast cancer | Many women are worry that they have breast cancer due to their blood test show high CA 15 – 3 score. So, what is CA 15-3 and is its increase the sign of breast cancer? |
| A008 | Breast cancer patient: Cancer cells develop faster in the case of hard tissue | According to a recent published research on Biomaterials, women with thicker and harder breast tissue will have higher risk of breast cancer, due to thick breast tissue provide the condition for cancer cells to attack normal cells |
| A009 | How can fish help prevent breast cancer? | Breast cancer emerges when the cells in breast start developing uncontrollably. Some of the symptoms of breast cancer include having lumps in breast, nipple fluids and changes of shape or structure of the nipple or breast. |
| A010 | Breast cancer among young people: Do you know how to protect yourselves? | In all countries, the average age of getting breast cancer is 60 – 65 years old. However, according to experts, in Viet Nam, the average age of getting breast cancer is only 40 – 50 years old. In some case, the patients were diagnosed when they are still very young. |
| A011 | Early detection of breast cancer through self-check at home | Thanks to periodically breast checking at home by touching, Mrs. T.T.N (63 years old, Ha Noi) was able to find a breast tumor at phase I – the early stage of breast cancer. After having an operation to remove the tumor, preserve the breast combine with medicine, her condition is now stable, and no cancer cell is found anymore. |
| A012 | Breast cancer detection: Experiment cancer test using urine sample | A Japanese company is ready to implement the first experiment on cancer diagnosis using urine sample, allowing better screening of this fatal disease |
| A013 | What do women need to do in order to prevent breast cancer? | Breast cancer is the most common kind of cancer among women, which takes away thousands of lives annually. According to the doctors, prevention is always better than treatment, and at each age, women need to take specific prevention methods |
| A014 | Breast cancer treatment using radiotherapy: More beneficial than harmful | Many breast cancer patients said they’ve heard scary stories regarding radiotherapy, however, in fact their experiences were much better, several recent researches mentioned. |
| A015 | 6 simple ways to reduce the risk of breast cancer | According to the US Cancer Association, 1 in every 8 women has the risk of getting breast cancer in life. However, by implementing the following positive activities, the risk of getting breast cancer will be reduced significantly |
| A016 | What are good exercises for breast cancer patients? | Research has found how practicing aerobic and bodyweight exercises may prolong the life expectancy of people who were successfully cured. |
| A017 | First medicine for women who acquired breast cancer from genetic mutation | FDA had licensed the first medicine for late stage breast cancer due to genetic reason similar to the actress Angelina Jolie |
| A018 | 90% of men are surprised to have breast cancer | Almost 90% of the men who were diagnosed with breast cancer by doctors were quite surprised to get this disease |
| A019 | Birth control pills increase the risk of breast cancer by 20% | According to a new research from Sweden, the uses of hormone-based contraceptives such as birth control pills may increase the risk of breast cancer by 20% |
| A020 | 02 more medicines for metastatic breast cancer treatment are implemented in Viet Nam | 02 new medicines targeting HER2-positive metastasis breast cancer: pertuzumab and trastuzumab emtansine (T-DM1) are officially used in Viet Nam |
| A021 | Breast cancer may return after 20 years | According to researchers, breast cancer may “lay low” and recur after 20 years unless the patients maintain the use of medicine to prevent it. |
| A022 | Treatment for advanced stage breast cancer: There are cases survive for more than 4 years | According to Dr. Le Thanh Duc, Director of the 5th Internal Medicine Department (National Cancer Hospital), the life expectancy of breast cancer patients has improved in the last 10 years |
| A023 | Would breast cancer become a burden of Viet Nam | According to Dr. Tran Nguyen Ha, Director of 4th Internal Medicine Department, Hochiminh City Cancer Hospital, breast cancer is in the top 5 most common cancer among Vietnamese women and is predicted to soon take the lead position, as comparing to other countries, the current prevalence is low, but have the trend to rise quickly. |
| A024 | 20 stars who “battled” breast cancer | From the prevention campaign by Lesley Murphy to Rita Wilson’s call for other women from her own experiences, listen to the stars sharing their stories of overcoming the disease. |
| B003 | Chance for breast cancer screening, consultation by professor from Singapore | Only one time on 24/7, Prof. Dr Mikael Hartman will come to the Singapore - Viet Nam Cancer Clinic to provide counselling and screening and treatment. |
| B004 | The Singaporean Professor, Doctor who treated hundreds of breast cancer patients | Dr. Mikael Hartman used to drive 25.000km by motorbike from Singapore to Sweden to raise a fund and awareness on breast cancer in Asia. |
| B006 | Female nurse has her breasts removed after 9 family members got cancer | 24-year-old Esther Taylor is among the youngest British women to have surgery to remove her breast in order to prevent breast cancer |
| B010 | Treating breast cancer without chemotherapy | A research on more than 10.000 breast cancer patients in the U. S has pointed out that sometimes chemotherapy is unnecessary, expensive, all the while causing serious side-effects. |
| B014 | Breast cancer patient becomes photo fashion model | Designer Li Lam invited many women to wear her dresses in order to inspire people to live positively |
| B015 | 10 years having regular health screening, yet didn’t know having got breast cancer | Obsessed and worried of old-age diseases, Mrs. Mai in District 7, Ho Chi Minh City regularly had periodical health check. Yet breast cancer still caught up to her unexpectedly |
| B017 | TVB star’s grief over inability to have children due to breast cancer | Dreaming of bearing children for her husband, still Lam Thuc Man had to give her dream up to get treatment |
| B026 | Signs of breast cancer on body that men should pay attention | The symptoms of breast cancer in men are similar to women: finding a lump, inverted nipple, fluid, abnormal breast development... |
| B029 | Breast removed due to doctor mistook test result with breast cancer patient’s | 46 years old Maryam Yazdany (Britain) are suffering from pain and stress after having one of her breast removed as the hospital mistakenly diagnosed her with breast cancer |
| B030 | Contraceptives increase the risk of getting breast cancer | Hormone-based contraceptives such as daily pill and implant may increase the risk of getting breast cancer among women by 38%. |
| B031 | Women who get breast cancer earlier in life may have high risk of recurrence | Young women who get breast cancer usually carry the mutated BRCA1/2 gene, and will have higher risk of getting breast cancer on the other breast than others. |
| B032 | Breast cancer may recur after 15 years | Scientists have found that breast cancer may recur and spread after 15-20 years since its declination. |
| B033 | 3-year-old child help aunt discover cancer by chance | Had Freddy not unexpectedly hit his aunt’s chest, 46-year-old Michelle Brown (England) wouldn’t have found the breast tumor and gotten treatment in time. |
| B034 | New medicine helps lengthen the life of breast cancer patients in Viet Nam | The Ministry of Health has just licensed 02 new medicines to treat HER2-positive metastasis breast cancer, in turn helps increasing patients’ lives by 5 years. |
| B036 | Peelings on skin may be signs of rare breast cancer | Starting with a peeling with the size of a rice on nipple, the wound took long time to heal and started to spread, Mrs. Ninh was diagnosed with breast paget. |
| B037 | Discover 72 new mutations leading to breast cancer | Scientists have just discovered 72 more mutations related to breast cancer, increasing the number of high-risk mutations to almost 180. |
| B039 | Wrong breast cancer diagnosis made woman lose both breasts | Elisha Cooke-Moore (USA) never thought she would be in such an awkward situation. Having been informed that she has genes causing cancer, the 36-year-old mother excepted to have her uterus and both her breast removed. Only afterward that she found that she was completely healthy. |
| B040 | 500 women participated Singapore-standard breast cancer screening | Almost 500 women participated in the campaign “Adore your bust, choose early screening” organized by Singapore-Viet Nam Cancer Clinic in Ho Chi Minh City |
| B042 | Charmed actress cried receiving chemotherapy for breast cancer | "I was so sick, I felt like losing myself ", actress Shannen Doherty spoke of the first time receiving breast cancer chemotherapy. |
| B043 | 8 hospital provides free breast cancer screening for patients | About 10.000 women from 40 years old were provided screening, breast ultrasound and mammography if suspected of cancer. |
| B044 | Hair dyes are suspected to cause breast cancer | A research in England has found that regular hair dyeing would increase the risk of getting breast cancer among women. |
| B045 | Angelina Jolie’s doctor shared 10 tips on breast cancer prevention | Dr. Kristi Funk advises women to eat more cruciferous vegetables, fruits with high oxidants, drink soy milk … in order to prevent breast cancer. |
| B047 | From a wart on breast, a woman discovered her breast cancer | Mrs. Tien found a small wart on her left breast when performed self-examination. When she came to hospital for biopsy and was diagnosed with breast cancer. |
| B048 | 8 simple methods to prevent breast cancer | Breast feeding, maintain weight, proper diet, have genetic examination if there are relatives with breast cancer … will help women to reduce the risk of getting the disease. |
| B049 | 4 common misunderstandings about breast cancer in women | Many people wrongfully assume that the larger the breast the easier to get cancer, or breast tension before menstruation is the sign of breast cancer. |
| B050 | Man breast swollen to weight 4kg due to breast cancer | Having an ulcer on the left breast, Mr. Quyen thought that was caused by insect, was diagnosed with breast cancer after having it checked. |
| B051 | Breast cancer battle diary of a mother | 32-year-old Melissa Thompson (USA) shares her story in order to inspire people with the same situation. |
| B052 | Breast cancer may spread to other body parts | Cancer cells may spread to bone, liver, lung, brain through blood veins and pea-shaped lymph nodes |
| B054 | Workaholic woman overcame breast cancer twice in 16 years | Having her right breast removed at 33, 16 years later, cancer cells spread to her left breast, however, not once that Mrs. Hoang Anh had the intention to give up. |
| B055 | 5 ways to keep positive after breast cancer treatment | Practice exercises at least 30 minutes a day; have a good diet; stay positive... are the best remedies for health. |
| B056 | Girls with precocious puberty have higher risk of breast cancer | If the girl starts her period when she is 8, parents better take their daughter for a health screening |
| B057 | Breast dents warn risk of breast cancer | The tumor is an accurate factor for breast cancer diagnosis, some changes of the body such as dents on breast may also be the warning sign of the disease. |
| C001 | Miraculous fruit that eliminates breast cancer cells | Researchers from Texas, USA have found that the extracts from peaches can kill cancer cells, ever in the case of the most “problematic” ones. |
| C002 | Good habits effectively prevent breast cancer any women can do regularly | Breast cancer is a dangerous disease among women. However, there are daily activities that may help reducing the risk of getting its. |
| C003 | Women who dye hair regularly have higher risk of getting breast cancer | Medical Professor Kefah Mokbel from Princess Grace Hospital, London recently has performed a study and concluded that women who dye hair regularly have high risk of getting breast cancer. |
| C004 | Stay away from bread if you have breast cancer | A substance found in bread may obstruct or reverse the effect of breast cancer medicine. |
| C005 | Marvela responded to breast cancer prevention campaign | Recently, the cooking oil products Marvela A & D3 (under Marvela Viet Nam) at all supermarkets had put on pink ribbons. It is announced that this is a community activity through the cooperation between Marvela and Breast Cancer Network Vietnam in response to the call for breast cancer prevention in Viet Nam. |
| C006 | New treatment choice for female breast cancer patients | Exemestane is a new medicine to treat breast cancer, which is more effective than the now common medicine to treat metastasis cancer -Tamoxifen in preventing metastasis breast cancer among young women, as well as the ones who had surgery to inhibit ovarian function. |
| C007 | Treating breast cancer with CBB therapy | CBB therapy allows therapists to accurately target the position of the removed tumor, in order to prevent the radioactive rays to affect the remaining normal breast cells. |
| C008 | Wife accepts to wear diaper over breast to maintain husband life | Even though having metastasis breast cancer, but as her husband had a stroke, Mrs. Hien accepted to wear diaper over her chest to save the little money left to sustain her husband’s life. |
| C009 | What food breast cancer patients must avoid | Non-Communicable Diseases (NCDs) are resulted from unhealthy habits people may have. Breast cancer is among the most typical NCDs. |
| C010 | Women who regularly do these activities have higher risk of getting breast cancer | A recent research has found that women who dye hair regularly have higher chance of getting breast cancer than others |
| C011 | 350 free breast cancer screening chances at The Garden | On 9/10/2017, The Garden Mall shall cooperate with Hong Ngoc General Hospital to give away 350 chances of free breast screening and ultrasound for women above 30 years old from 12 - 20/10/2017. |
| C012 | The foods which are cancer’s “bane” and good for patients | 8 meals mentioned in the following article should be remembered if you want to improve your immune system and prevent breast cancer. |
| C013 | What should be known about breast cancer? | Breast cancer occurs mostly among women, but men may also get the disease. Breast cancer is the most common type of cancer as well as the leading cause of death among women in industrial countries |
| C014 | Inexpensive traditional remedies for breast cancer | Breast cancer is a dangerous and common disease among women, which may threaten the lives of the patients as well as their survivability. Eastern Medicine and traditional medicine has several remedies for breast cancer treatment from available ingredients |
| C015 | What cause leading to breast cancer? | Breast cancer is the type of cancer developed from milk duct epithelium or the lobes at the other end of the breast, after which they will multiply and spread directly to other tissues or parts of the body. |
| C016 | Blue ganoderma cures breast cancer | Blue garnoderma is a valuable herb to cancer patients: breast cancer, stomach cancer, liver cancer, the rare substances found in garnoderma help eliminating cancer cells and protect healthy cells. |
| C017 | Completely cured of breast cancer thanks to Crinum Latifolium | Depend on the stage of disease, the patient’s health, other related factors, the doctor shall prescribe the most suitable treatment for breast cancer patients. |
| C018 | What should you refrain from when having breast cancer? | Diet also plays an important role in parallel to the treatment in treating breast cancer. Hence, it is worth concerned what breast cancer patients should refrain from eating. |
| C019 | “Naïve” mistakes about breast cancer | Many people still think that breast cancer is mostly due to genetic, or young women wouldn’t get this disease, or getting breast cancer means having a tumor … All of these are wrongful thoughts of such a dangerous disease. |
| D002 | Screen for breast cancer when turning 40 | It is the message emphasized at the launching of the Action month for breast cancer prevention by the Ministry of Health, Cancer patients support fund – Bright future and other partners on October 14 on Hanoi Opera House Square |
| E001 | 15 recommendations to prevent breast cancer | Breast cancer is the most common and the leading cause of mortality among women in industrial countries. This is a complicated disease, which over the years there are many studies regarding its causes and treatment. However, it is needed to detect breast cancer early through the screening of normal women. In France, for every 10 women, one will have breast cancer, but luckily, there are methods to help preventing the disease such as good diet, exercises … Following is 15 advices for breast cancer prevention |
| E002 | Signs of breast cancer | I am 45 years old. Recently I found in my right breast, near my arm pit, a small hard stable tumor, it hurts when pressed. |
| E003 | Eating cruciferous vegetables helps decreasing the risk of breast cancer | Cruciferous vegetables mentioned here include green cauliflower, white cauliflower, kale, cabbages. In recent years, the world is focusing on these vegetables, mainly for their surprising health benefits. |
| E004 | Ha Tinh: provide free breast cancer screening for almost 600 women | On morning 23/6/2018, Ha Tinh Provincial General Hospital in cooperation with the Young Doctor Association and Pharmaceutical Association, Center for Nuclear Medicine and Oncology of Bach Mai Hospital provides free breast cancer screening, counselling. |
| E005 | Helpful meals – remedies for breast cancer treatment | In Eastern Medicine, breast cancer is called “Nhu Nham”. In the medical documents from Yuan Dynasty, they described diseases “Đố nhũ”, “Hạch bì tương thân” with typical symptoms similar to breast cancer. |
| E006 | Notes regarding the diet of breast cancer patients | There is no universal solution regarding the diet for all breast cancer patients, said Megan Morrison, Nutrition Specialist at Princess Margaret Cancer Center, Toronto |
| E007 | Warning signs of breast cancer in men | Breast cancer is a common type of cancer among women. However, men should also be wary of this type of cancer, eventhough the chance of getting breast cancer among men is extremely lower (about 1%) |
| E008 | How does cabbage help prevent breast cancer? | Breast cancer is among the most dangerous diseases. Early detection and proper treatment is crucial in treating this disease |
| E009 | Breast cancer symptoms on mammogram may increase risk of getting breast cancer | A recent study shows that women who have abnormal symptoms in breast during periodical screening have higher chance of developing breast cancer before the next screening |
| E010 | Breast dimples: Be wary of breast cancer | An Australian woman, Kylie Amstrong took a photo of her breast and uploaded it to Facebook speaking about a strange symptom that was happening to her: a dim breast dimple |
| E011 | Breast cancer, cervical cancer spares no ones, men or women | According to Dr. Doan Huu Nghi – Formal Director of National E Hospital, Formal Deputy Director of Vietnam National Cancer Hospital: Annually, there would be 10-20 people among 100.000 under-30 women diagnosed with cervical cancer. Whether men or women, it is still possible to get gynaecological infections, breast cancer, cervical cancer. However, there diseases can be prevented and treated by early diagnosis and timely treatment |
| E012 | Soy bean for breast cancer patients: Good or bad | Rumours said that eating soy beans will increase the risk of getting certain types of cancer, especially breast cancer as estrogen is related to the development of estrogen-sensitive cancer such as breast cancer |
| E013 | Dangerous warning symptoms of breast cancers people need to know | Breast cancer can be prevented if detected early. Follow are the dangerous symptoms of breast cancer that you need to know |
| E014 | Foods that help prevent breast cancer | Breast cancer is a complicated disease which relates to various factors. Some cannot be controlled such as gender, age, genetic |
| E015 | 80% of breast cancer cases directly related to living conditions | Currently, the direct causes of breast cancer are still not found, however, a tight connection between external, internal factors and breast cancer’s hormones has been discovered |
| E016 | Acupuncture may help relieving the pains from breast cancer treatment | Several medicines used in breast cancer treatment may cause arthritis, however a recent study found that acupuncture may help reduce this side effect |
| E017 | New medicine for breast cancer may be beneficial for women | A recent clinical trial had found a new medicine for the standard regiment which slows the development of breast cancer among younger women |
| E018 | Viet Nam receives 02 more medicine targeting HER2-positive metastasis breast cancer | At the conference “New leap in treaing HER2-positive metastasis breast cancer”, leading experts from all over the country had discussed, updated information related to pertuzumab and trastuzumab emtansine (T-DM1) – 2 new medicines in the targeting treatment for metastasis breast cancer endorsed by the Minitry of Health |
| E019 | Can breast cancer be treated without surgery? | There are many treatments for breast cancer such as, surgery, chemotherapy, radiotherapy, hormonal therapy and targeting therapy |
| E020 | Quang Ninh: A woman with rare breast cancer is saved | Doctors at Vietnam Switzerland Hospital in Uong Bi, Quang Ninh had discovered and successfully treated a rare case of breast cancer in 1 patients – which only take 1-4% among all types of breast cancer. Currently, the patients is stable, can eat, drink, walk normally |
| E021 | Yakult supports free breast cancer screening for 10,000 women | This program is among the chain of activities to support breast cancer screening among women and mothers at the hospitals in Ho Chi Minh city, Hanoi and Da Nang. The main activities started on 14/10/2017 |
| E022 | Ha Tinh Young Doctor Association: Free breast cancer screening for 500 women | Ha Tinh Young Doctor Association had cooperated with the National Cancer Hospital and Provincial General Hospital to organize a session for screening, counselling for women at the city, or from Ha Tinh and areas near there |
| E023 | Free ultrasound, mammography for breast cancer early detection | In response to the campaign “We care for her – For women and the future” by the Ministry of Health and Fund to support cancer patients – Bright future. The Center for Nuclear medical and Cancer, Bach Mai hospital will accompany this program in order to implement the program “Screen for Breast cancer when turning 40” |
| E024 | Have yourselves screened for breast cancer right away if you find a lump | Appearance of a lump or tumour in the breast is common among women at reproductive age. This symptom may relate to normal breast disease such as breast hyperplasia, … However, it could also be the most common symptom of breast cancer |
| E025 | Great leaps in breast cancer treatment in Viet Nam | The rate of getting breast cancer in our country is increasing as the age of patients is getting younger. However, disease prevention has achieved significant achievement over the last 10 years |
| E026 | After turning 40, early screening for breast cancer may allow up to 80% of being treated | On October 15, within the World Breast Cancer Prevention Month, Ministry of Health, Fund to support breast cancer patient – Bright future will launch the campaign: “Breast cancer screening after turning 40” at Hochiminh City Medical and Pharmaceutical Hospital. The launching of the project is also honoured to have the present of Assoc.Prof. Dr. Nguyen Thi Kim Tien, Minister of health |
| E027 | Screen for breast cancer after turning 40 | It is the message emphasized at the launching of the Breast Cancer Screening campaign in the frame of Action month for breast cancer prevention by the Ministry of Health, Cancer patients support fund – Bright future and other partners on October 14 on Hanoi Opera House Square |
| E028 | 8 signs of recurrence breast cancer | At the early stage of breast cancer, surgery and chemotherapy, radiotherapy will be applied in the treatment |
| E029 | Using leaves for breast cancer treatment: Cost money for more diseases | Swollen breast, ulcers, increased tumor, metastasis due to using leaves |
| E030 | Breast feeding reduces the risk of breast cancer for both the mother and child | Not only reducing the risk of breast cancer for the mother, breast feeding also help to reduce the risk of breast cancer in the future of her children. (According to the report of Cancer Institute). |
| E031 | FDA endorsed the new medicine to treat recurrence breast cancer | The US Food and Drug Administration had just endorse Verzenio to be used to treat HER2-negative and HR-positive metastasis breast cancer patients |
| E032 | New promising medicine for breast cancer treatment | A clinical trial had found that Z-endoxifen safely and effectively shrink the tumor among women who has ER-positive metastasis breast cancer |
| E033 | 6 simple methods to prevent breast cancer | Breast cancer í the most common cancer among women with a high death rate. In Vietnam, for every 10 women, 01 will have a chance of developing breast cancer, which is rather high. Luckily, scientists also found simple methods which may help girls to effectively prevent breast cancer |
| E034 | New medicine brings hope for breast cancer patients | Scientists have identified a molecule which may help treating breast cancer, bringing hope to patients who have already gain resistance over common treatment therapy |
| E035 | Using antiperspirants is safe during breast cancer treatment | In contrary to the worry of many doctors, a recent research found that it is possible to use antiperspirant among breast cancer patients who are receiving radiotherapy |
| E036 | Eating vegetables reduces the risk of breast cancer | Millions of women in the world are using HRT in combination with estrogen and progestin in order to reduce the discomfort during menopause |
| F001 | Shuttlecock belle Huyen Trang passed away from cancer | After a long struggle with cancer, shuttlecock belle Nguyen Thi Huyen Trand just could not overcome her fate |
| F002 | 7 best methods to prevent breast cancer | There are simple advices yet help you reduce the risk of getting breast cancer. The disease occurs when the cell in breast started to develop uncontrollable. |
| F003 | Riding motorbikes for breast cancer prevention | Nnenna Samuila and Jeminat Olumegbon started the D’Angels group in 2009, after being refused from participation by male motorbike groups in Lagos. In 2010, the 2 friends took a 617-km journey on their bike from Lagos to the Southern city, Part Harcourt. This is the moment that would change men’s perception on their ability |
| F004 | 24 years old nurse had her breasts removed after 9 relatives got cancer | Esther Taylor, a 24-year-old pediatrics nurse from Preston, Lancashire county, England has become one of the youngest women in UK to have surgical to remove breasts, even though she doesn’t have the BRCA gene which cause the deadly cancer. |
| F005 | High-risk of breast cancer for lacking vitamin D | People who lack vitamin D have higher chance of getting breast cancer, colon cancer, prostate cancer, cardiovascular diseases than others. Several studies in Vietnam found that, the lack of Vitamin D is more common among women than men |
| F006 | Shine like a star even without one’s hair and breasts | Vietnam Breast Cancer Network cooperated with LiLam Designer to launch an album in honor of breast cancer women with the name “Always a woman to me” on Mother’s day 13/5 |
| F007 | A medicine which makes the tumors glow allowing easier diagnosis of breast cancer | Scientists from Michigan University (USA) had discovered a pill which makes the tumor glow under infrared light and let doctors detect, diagnosis breast cancer earlier, easier, with higher accuracy and lower risk for patients |
| F008 | Artificial mole helps early detection of breast cancer | Scientists announced that artificial mole with the ability to diagnosis early 4 types of cancer including prostate cancer, breast cancer, lung cancer and colon cancer, may become real in the next 10 years |
| F009 | The miraculous journey of a female journalist overcoming breast cancer | To Ms. Cam Bao, even after having to give up her hair, breasts, yet never did she give up her faith to live, never stop spreading her optimism to other patients |
| F010 | Wearing pink ribbons in response to breast cancer prevention campaign | In Vietnam, breast cancer had surpassed cervical cancer to become the most common and the most fatal. It is estimate that annually, there is an average of 11.000 cases of breast cancer in Viet Nam |
| F011 | Breast cancer takes 21% of the cancer cases among women | This information was provided at the Conference “A new leap in treating HER2-positive metastasis breast cancer”, organized by National Cancer Hospital in cooperation with the Representative Office of Hoffmann La Roche on November 11 in Hanoi |
| F012 | Identify 72 genetic mutations which may increase the risk of breast cancer among women | According to a recent study on the journal Natural and Genetic issue on October 23, scientists had discovered 72 genetic mutations which increase the risk of getting breast cancer among women |
| F013 | “Pink fighter” to prevent breast cancer | They are the people who eventhough have been fighting the disease, but still optimistic. Cancer actually becomes a trial to make them stronger, more beautiful and allow them to live a more meaningful life |
| F014 | 11 groups with highest risk of getting breast cancer | Women who have family members with breast cancer, taking many contraceptive pills, early puberty or late menopause, have higher risk of developing breast cancer than others |
| F015 | Have breast cancer screening after turning 40 | This campaign is launched by the Fund to support cancer patients – Bright future (MOH) from 14/10 to 11/11 in large specialized hospitals in 3 cities – Hanoi, Hue and Ho Chi Minh City |
| F016 | 14 women show their naked breasts full of scars to raise awareness on breast cancer | 14 women volunteered to become models for photographer Ami Barwell in an album as part of the “Stand Up To Cancer” campaign during the month for raising awareness about breast cancer |
| F017 | Breast cancer and cervical cancer screening for women above 35 | Ho Chi Minh City Medical and Pharmaceutical University cooperated with Fund To Support Cancer Patients – Bright future (MOH) to organize the “Early screening and diagnosis of Cancer” program on 13.00 of 23/9, 30/9 and 7/10 |
| F018 | Leading cancer among women: Breast cancer | On average, everyday our country has 40 new cases of breast cancer, and 16 pass away from the disease. This is the most common type of cancer among women |
