# Supplementary material for: Breast Cancer messaging in Vietnam: an online media content analysis
Source: BMC Public Health. 2020 Jun 19;20:966. doi: 10.1186/s12889-020-09092-8 (PMC7304170; doi:10.1186/s12889-020-09092-8)
Supplement: Supplementary file 2 — Additional file 2. Other supplementary information provided was 1. data collection matrixes and 2. image analysis matrix. [file 12889_2020_9092_MOESM2_ESM.doc]

# Coding Sheet: Breast Cancer in Vietnam

# coder initials …………………..

| 2 **Date of Article** | | | | | |
| --- | --- | --- | --- | --- | --- |
| Day Month Year | | | | | |
|  |  |  |  |  |  |

|  | | 1  **Article ID** | | | |
| --- | --- | --- | --- | --- | --- |
|  | Letter (Source) | | NUMBER | NUMBER | NUMBER |

| 3 | **Headline** (full) |
| --- | --- |
|  |  |
|  |  |
|  |  |

| 4 Link (if online) |
| --- |
|  |

| **5 Rate headline** |  |
| --- | --- |
| Alarmist (likely to increase reader anxiety, emotive language) | 1 |
| Reassuring (likely to reduce reader anxiety) | 2 |
| Neither /Nor (tends to be factual, a statement or bland language) | 3 |

| **4 Location of article (if in paper)** |
| --- |
| **Front Page 1** |
| **Not Front Page 2** |

| **6 Focus of Article** |  |
| --- | --- |
| Cancer (inc. breast cancer) | 1 |
| Breast cancer specifically | 2 |

| **8 Lead Paragraph** |  |
| --- | --- |
|  | |

| 7 **Word Count** | | | |
| --- | --- | --- | --- |
| NUMBER | NUMBER | NUMBER | NUMBER |

|  | **Are any of the following expressed in the text?** | YES | NO |
| --- | --- | --- | --- |
| Problem definition | States breast cancer rates | 1 | 2 |
| Problem definition | Mentions breast cancer incidence as increasing | 1 | 2 |
| Problem definition | Correctly mentions risk factors related to breast cancer | 1 | 2 |
| Problem defintion | Incorrectly identifies risk factors (eg. karmic beliefs/other) | 1 | 2 |
| Problem defintion | Casual Factors linked to Westernisation (of/ eg. diet) | 1 | 2 |
| Problem definition | Correctly identifies symptoms of breast cancer | 1 | 2 |
| Problem definition | Incorrectly identifies symptoms of breast cancer | 1 | 2 |
| Problem definition | Encourages women to speak to their healthcare provider if they experience symptoms | 1 | 2 |
| Problem definition | Mentions treatment options for women with breast cancer in a **reassuring** tone (ie treatment can be curative) | 1 | 2 |
| Problem definition | Mentions treatment options for women with breast cancer in an **alarmist** tone (‘deforming’, hair loss) | 1 | 2 |
| Problem definition | Mentions economic challenges for women with a breast cancer diagnosis | 1 | 2 |
| Problem definition | Mentions financial catastrophe related to breast cancer treatment | 1 | 2 |
| Problem definition | Mentions breast cancer as a burden to the health system | 1 | 2 |
| Social challenges | Mentions social challenges related to breast cancer, such as stigmatisation or experiencing discrimination | 1 | 2 |
| Social challenges | Mentions breast cancer as a cosmetic / beauty problem | 1 | 2 |
| Social challenges | Mentions women with breast cancer experiencing relationship breakdown with intimate partners | 1 | 2 |
| Interventions | Mentions Government interventions to support women with breast cancer | 1 | 2 |
| Interventions | Mentions social support networks for women with breast cancer | 1 | 2 |
| Interventions | Mentions arts-interventions to raise awareness about symptoms of breast cancer | 1 | 2 |
| Interventions | Mentions arts-interventions to reduce stigmatisation of women with breast cancer | 1 | 2 |
| Interventions | Mentions traditional medicine as viable alternative to biomedical interventions | 1 | 2 |
| Interventions | Mentions screening options for breast cancer | 1 | 2 |
| Interventions | Describes what is involved in a screening procedure | 1 | 2 |
| Interventions | Provides information on where and how to access screening services | 1 | 2 |
| Social challenges | Blames women for their diagnosis (karmic causes) | 1 | 2 |
| Social challenges | Blames women for their diagnosis (for delaying speaking to a healthcare provider) | 1 | 2 |
| Problem definition | Criticises the health system and healthcare providers for lack of systematic capacity to respond to breast cancer | 1 | 2 |
| Interventions | Advertises pharmaceutical interventions or products for breast cancer | 1 | 2 |
| Other |  | 1 | 2 |
| Other |  | 1 | 2 |
| Other |  | 1 | 2 |
| Other |  | 1 | 2 |

| 10 | **Qualitative Analysis (1)**  **(Content and quotations included within the article)** | |
| --- | --- | --- |
| Stakeholder Position ‘VOICE’ (journalist, patient, survivor, healthcare provider, government official etc., and information on organisation stakeholder represents if applicable) | | Key Quotes |
|  | |  |
|  | |  |
|  | |  |
|  | |  |
|  | |  |

| 11 | **Researchers Reflections** |
| --- | --- |
|  | |

IMAGE or VIDEO Information

1. Image/Video description (duration, context):

2. Position of Image (top of article, included within article, end of article)

3. Position size: Full page, half page, quarter page, smaller than a quarter page

4. Include copy of image if possible:
